# Form and function of the human and chimpanzee forefoot: implications for early hominin bipedalism

**DOI:** 10.1038/srep30532

**Published:** 2016-07-28

**Authors:** Peter J. Fernández, Nicholas B. Holowka, Brigitte Demes, William L. Jungers

**Affiliations:** 1 Department of Anatomical Sciences, Stony Brook University, Stony Brook, NY 11794, USA; 2 Department of Human Evolutionary Biology, Harvard University, Cambridge, MA 02138, USA; 3 Association Vahatra, BP 3972, Antananarivo 101, Madagascar

## Abstract

During bipedal walking, modern humans dorsiflex their forefoot at the metatarsophalangeal joints (MTPJs) prior to push off, which tightens the plantar soft tissues to convert the foot into a stiff propulsive lever. Particular features of metatarsal head morphology such as “dorsal doming” are thought to facilitate this stiffening mechanism. In contrast, chimpanzees are believed to possess MTPJ morphology that precludes high dorsiflexion excursions during terrestrial locomotion. The morphological affinity of the metatarsal heads has been used to reconstruct locomotor behavior in fossil hominins, but few studies have provided detailed empirical data to validate the assumed link between morphology and function at the MTPJs. Using three-dimensional kinematic and morphometric analyses, we show that humans push off with greater peak dorsiflexion angles at all MTPJs than do chimpanzees during bipedal and quadrupedal walking, with the greatest disparity occurring at MTPJ 1. Among MTPJs 2–5, both species exhibit decreasing peak angles from medial to lateral. This kinematic pattern is mirrored in the morphometric analyses of metatarsal head shape. Analyses of *Australopithecus afarensis* metatarsals reveal morphology intermediate between humans and chimpanzees, suggesting that this species used different bipedal push-off kinematics than modern humans, perhaps resulting in a less efficient form of bipedalism.

During the evolution of bipedalism the hominin foot underwent a number of dramatic changes that converted it from a prehensile grasping organ to a strong propulsive lever. Among the features affected by these changes, those of the forefoot region have received much attention in comparative morphology studies due to their role in the push-off phase of bipedal walking^1^,^2^,^3^,^4^,^5^,^6^,^7^,^8^. The extant great ape forefoot is characterized by an abducent opposable hallux and metatarsal (MT) heads that are plantarly oriented in sagittal profile and dorsally narrow^1^,^9^,^10^,^11^,^12^. Additionally, the phalanges are long and curved, enabling them to resist the bending strains incurred during pedal grasping^13^. In contrast, the human forefoot is characterized by a permanently adducted hallux that is incapable of opposing the other digits, and more dorsally oriented and mediolaterally broad metatarsal head articular surfaces. The phalanges of modern humans are short and straight, and the proximal phalangeal bases demonstrate dorsal canting^2^,^14^,^15^,^16^,^17^.

The interfaces of the metatarsal heads and proximal phalangeal bases, the ellipsoidal metatarsophalangeal joints (MTPJs), allow plantarflexion for pedal grasping and dorsiflexion during push off. The dorsally oriented MT heads in the human forefoot are thought to increase the dorsiflexion range of motion (ROM) at the MTPJs^18^,^19^ by providing more dorsal articular surface area on which the proximal phalangeal base can slide. In humans, dorsiflexion at the MTPJs causes tightening of the plantar aponeurosis, which is a thick band of ligamentous tissue that originates from the calcaneal tuberosity and inserts distally on the proximal phalangeal bases. Tightening of this structure acts as a “windlass mechanism”^20^,^21^ that raises the longitudinal arch, providing midfoot stability and facilitating propulsive force production at push off 22. The plantar aponeurosis is only weakly developed in great apes, and consequently these species are believed to lack a windlass mechanism^1^,^11^. Additionally, the more plantarly oriented MT head in great apes is hypothesized to increase plantarflexion ROM for arboreal pedal grasping^8^,^18^,^23^,^24^ at the expense of dorsiflexion ROM during push off on terrestrial substrates^18^,^19^,^24^.

The mediolaterally expanded dorsal MT head in humans has been argued to enhance joint stability under increased loads during push off from highly dorsiflexed MTPJs^8^,^23^,^25^,^26^,^27^. Dorsal mediolateral expansion of the MT heads also allows for close-packing of the MTPJs by tightening the collateral MTP ligaments when the joints are in dorsiflexed postures^27^,^28^,^29^. In the close-packed position the appositional surfaces of the MT head and proximal phalangeal base are maximized, and the tightening of collateral ligaments limits joint motion outside of the sagittal plane, increasing joint stability when maximum joint congruency is achieved^23^,^28^. This same mechanism is hypothesized to close-pack the great ape MTPJs in plantarflexion for increased stability during pedal grasping.

These differences in human and great ape MTPJ functional morphology have been used in analyses of fossil hominin pedal remains to assess the degree to which early hominins were capable of pushing off in a manner similar to humans during bipedal locomotion. Typically, human-like morphology is interpreted as indicating a modern human-like push off, whereas ape-like features are taken to indicate less derived push-off mechanics. For instance, the presence of dorsally oriented MT heads has been interpreted as indicative of human-like bipedalism in *Australopithecus afarensis*^19^, but others have argued that the relatively narrow dorsal breadth of the MT heads in this species suggests a push off that was different from that of modern humans^26^. Conflicting interpretations of MT head morphology such as these can only be resolved by combining comprehensive shape analyses with quantitative foot motion data and by establishing functional links between joint surface shape and joint motion. Three-dimensional geometric morphometric (3DGM) methods allow for the quantification of shape differences in bony elements between individuals or among species. Recently, Fernández *et al*.^8^ demonstrated the power of 3DGM to distinguish MT head morphology among primate taxa that possess different locomotor repertoires. Expanding on this approach, we here relate the shape differences between species quantified in that study to differences in joint function in chimpanzees and humans during terrestrial locomotion.

The extent to which the MTPJ motion of humans and other great apes differs during locomotion has not been comprehensively established. In the only study that quantified MTPJ motion *in vivo* in a non-human great ape, Griffin *et al*.^3^ found significantly greater joint angles at push off in humans than in bonobos for the first two pedal rays. However, they were only able to analyze a small number of steps, and found some overlap between species in push-off joint angles. This ambiguity suggests that African ape joint kinematics may be somewhat similar to that of humans during push off in terrestrial locomotion, undermining the form-function relationship assumed for the hominoid MTPJs. To investigate this relationship more thoroughly, a larger sample of great ape steps must be analyzed, and all five MTPJs should be assessed to determine if interspecies differences hold up across the pedal rays. These data can then be compared to matching morphological data to determine whether current form-function assumptions for the metatarsal heads are in fact valid.

Recent works have incorporated 3-dimensional (3D) shape analysis methods to robustly discern functional signals in skeletal elements^30^,^31^,^32^, and the coupling of these data with accurate kinematic data will enable experimental testing of assumptions about MTPJ functional morphology. This design will facilitate the reconstruction of bipedal kinematics in extinct hominins from analyses of pedal fossils, thereby improving our understanding of the origins of bipedalism and human evolution. Pedal remains attributed to fossil hominins are known from a large temporal and geographic range that includes much of Africa and Eurasia and as far back as 4.4Ma. Nearly all of these taxa, including *Ardipithecus ramidus*, *Australopithecus afarensis*, *Homo erectus*, *Homo floresiensis*, and assorted South African hominin taxa display a mosaic morphology of human and ape-like features in the forefoot (e.g.^18^,^25^,^27^,^33^,^34^,^35^). Thus, a detailed understanding of how MTPJ function is related to joint anatomy is necessary to verify current hypotheses about hominin forefoot biomechanics and the evolution of bipedalism.

To this end, we collected 3D marker-based kinematic data for MTPJs 1–5 during bipedal and quadrupedal walking in chimpanzees (*Pan troglodytes*), and bipedal walking in humans (*Homo sapiens)*. We compared these data to landmark-based 3DGM data from the MT heads of all five pedal rays in these species to test the hypothesis that differences in human and chimpanzee MTPJ function during walking are reflected in morphological differences at their metatarsal heads. Following this hypothesis, we predicted that humans would use higher peak dorsiflexion angles than chimpanzees at all MTPJs, regardless of whether the latter walked bipedally or quadrupedally. To stabilize the MTPJs at higher dorsiflexion angles, we predicted that human MT heads would be more dorsally oriented and dorsally wider than the chimpanzee MT heads at all of the MTPJs. Additionally, we predicted that significant differences in peak dorsiflexion angles between MTPJs would reflect significant differences in metatarsal head morphology between rays within species.

Finally, we collected 3DGM data from fossil MT heads from rays 1–5 attributed to *A. afarensis* (Fig. 1). As previously discussed, different features of the *A. afarensis* MT heads have been used to argue either that this species walked with modern human-like kinematics^19^,^36^, or pushed off differently than humans during bipedal locomotion^23^. To address this debate, we assess the morphological affinities of *A. afarensis* MT heads in comparison to those of humans and chimpanzees using 3DGM. Our findings are then placed in the context of the relationship between MT morphology and MTPJ function, and used to draw conclusions about bipedal gait kinematics in *A. afarensis*.

## Results

### Chimpanzee and human MTPJ kinematics

We analyzed MTPJ motion in five steps per human subject (N = 25), ten bipedal steps per chimpanzee subject (N = 20), and five quadrupedal steps per chimpanzee subject (N = 10). In both species MTPJ dorsiflexion for all joints peaked in the final 20% of stance phase, just prior to liftoff (Fig. 2). Humans exhibited significantly greater dorsiflexion angles at all five joints than chimpanzees did during both bipedal and quadrupedal locomotion (Table 1; Fig. 3; Mann-Whitney U [MWU] test, *P* < 0.001 for all comparisons). The greatest disparity in joint angles occurred at MTPJ 1, where humans dorsiflexed their joints 36° more on average than chimpanzees. Moving laterally across MTPJs 2–5, both humans and chimpanzees exhibited a decrease in peak joint angles, although the gradient of this decline was steeper in chimpanzees. Peak MTPJ angles were similar in chimpanzees during bipedal and quadrupedal walking, with the only significant difference between locomotor modes occurring at MTPJ 3, where chimpanzees used 5° more dorsiflexion during quadrupedalism on average (Table 1; Fig. 3; MWU test, *P* = 0.001).

For both humans and chimpanzees, peak dorsiflexion angles were significantly different among the MTPJs (Table 2; Kruskal-Wallis [KW] test, *P* < 0.001 for all tests). For the human steps, MTPJ peak dorsiflexion angles were statistically indistinguishable among the first four joints, but MTPJ 5 had significantly lower peak dorsiflexion angles than all other joints (*post hoc* KW test, *P* < 0.001 for all comparisons involving MTPJ 5). Among the chimpanzee bipedal and quadrupedal steps, MTPJ 1 peak dorsiflexion angle was statistically indistinguishable from those of MTPJs 4 and 5, but significantly lower than those of MTPJs 2 and 3 (*post hoc* KW test, *P* < 0.001 for all comparisons). During bipedal walking, MTPJs 2 and 3 had significantly greater peak dorsiflexion angles than those of all other joints (*post hoc* KW test, *P* < 0.005 for all comparisons), but were statistically indistinguishable from one another. During quadrupedal walking, MTPJ 2 had significantly greater angles than all other joints except MTPJ 3, and MTPJ 3 had significantly greater angles than MTPJs 1 and 5 (*post hoc* KW test, *P* < 0.001 for all comparisons).

### 3D Geometric Morphometrics

For all MTs, shape variables PC1–PC 5 individually accounted for at least 5% of the variance in the data and were explored in more detail. Because PC1 and PC2 captured MTPJ articular surface morphology that was hypothesized to be related to dorsiflexion at push off 8, results for these two axes are reported in further detail. Morphometric data for MTs 2–5 were pooled in order to examine intraspecific MTPJ comparisons; pooling these data was possible because PC1 and PC2 capture the same shape differences for MT2–MT5 in humans, chimpanzees, and other catarrhines^8^. MT1 was analyzed separately due to homology constraints related to differences in ontogenetic development and gene expression of the hallux^37^.

For MT1, PC1 completely separated humans and chimpanzees (*post hoc* Tukey’s HSD, *P* < 0.001), whereas the groups overlapped on PC2 (Fig. 4a). PC1 (38% of variance) captured shape changes related to the overall dorsal versus plantar orientation of the MT head in sagittal profile as well as shape changes in plantar condylar morphology. Negative PC1 scores, as exhibited by human MT1s, indicate more dorsally oriented MT heads with broader, shallower plantar condyles. Positive PC1 scores, as exhibited by chimpanzee MT1s, characterize more plantarly oriented MT heads with deeply projecting plantar condyles flanked by steep, angular facets for articulation with the hallucal sesamoid bones; humans have dorsally wide MT1 heads, whereas chimpanzees have plantarly wide MT1 heads. PC2 (11% of variance) captured variance in the relative mediolateral breadth (robusticity) of the MT1 head. High PC2 scores indicate greater overall mediolateral robusticity of the MT head. There is no separation between species on this axis.

The pattern observed in MT2–MT5 was similar. PC1 (29% of variance) tracked two principal shape changes: epicondylar breadth and dorsal versus plantar orientation of the MT head relative to the epicondyles, which completely separated humans and chimpanzees (*post hoc* Tukey’s HSD, *P* < 0.001) (Fig. 4b). PC2 (12% of variance) captured overall mediolateral robusticity of the MT2–MT5 heads, with higher PC2 scores indicating greater relative mediolateral width and serving to separate medial from lateral MTs within species (Table 2). In humans, MT2 was significantly more mediolaterally robust than MT3–MT5, and MT3 was significantly more robust than MT4–MT5 (*post hoc* Tukey’s HSD, *P* < 0.001). The pattern found in chimpanzees was slightly different; MT2 was indistinguishable from MT3, but was significantly more robust than MT4–MT5 (*post hoc* Tukey’s HSD, *P* < 0.001; *P* < 0.001), MT3 was indistinguishable from MT4 but was significantly more robust than MT5 (*post hoc* Tukey’s HSD, *P* < 0.001), and MT4 was indistinguishable from MT5. Overall, MT2–MT5 PC2 scores and peak MTPJ dorsiflexion angles decrease from medial to lateral across the forefoot in both species.

**Figure 1:**
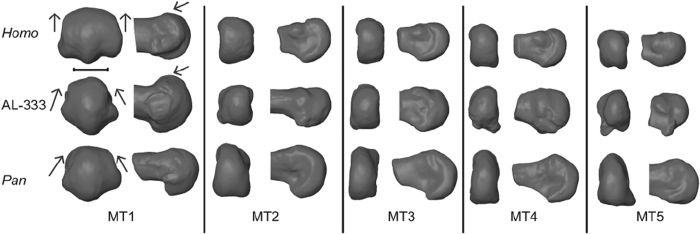
Comparative morphology of *Homo, Pan*, and *A. afarensis* (AL-333) metatarsals (MT1–MT5). Note that *Homo* is characterized by dorsal overlap of the distal articular surface onto the MT shaft and by wide flattening of the dorsal articular surface (arrows). AL-333-115 demonstrates *Homo*-like dorsal overlap but lacks mediolateral widening at the MT1, similar to the condition seen in *Pan*. Left column: distal view. Right column: medial view. Bar: 1 cm.

The A.L. 333-115 MTs fell within the human and chimpanzee ranges of variation on both PC1 and PC2 (Fig. 4 and Supplementary Fig. 2). The hallucal element, A.L. 333-115A, fell just within the chimpanzee range on PC1 and was well outside the range of modern human variation (Fig. 4a), but it also fell outside the range of chimpanzees on PC2. Although A.L. 333-115A exhibits marked dorsal doming, especially when viewed medio-laterally (Fig. 1), it does so in a way unlike modern humans. The *A. afarensis* MT1 head is expanded dorsally, but not mediolaterally widened as seen in *Homo*; rather, the dorsal MT head is narrow and rounded, similar to the chimpanzee condition. Thus, while A.L. 333-115A undoubtedly shows some human-like MT head shape characteristics, shape analyses demonstrate that the overall MT1 head shape of *A. afarensis* is most similar to extant *Pan*. This is immediately contrasted in A.L. 333-115B (MT2), which falls directly in the *Homo* MT2 morphospace (Fig. 4b). It demonstrates both the dorsal expansion and widening of the MT head seen in modern humans, and this is confirmed by its overlap with humans on both PC1 and PC2 to the exclusion of chimpanzees. A.L. 333-115C (MT3) falls intermediate between humans and chimpanzees on PC1, but overlaps with both species on PC2. A.L. 333-115D (MT4) is chimpanzee-like on both axes, and A.L. 333–155E (MT 5) is human-like on PC1, but overlaps both species on PC2. Overall, the *A. afarensis* forefoot is mosaic and unique in its morphology.

## Discussion

We hypothesized that differences in human and chimpanzee MTPJ function during terrestrial locomotion would be reflected in morphological differences at the metatarsal heads. When viewed in parallel, the kinematic and morphometric data collected in this study strongly support this hypothesis and both of our predictions. In support of the first prediction, humans exhibit greater peak dorsiflexion angles than chimpanzees at the MTPJs whether the latter walk on two or four limbs, which is reflected in distal MT articular surface morphology. The interspecific difference in MTPJ motion is driven by proximal hind limb angular motion; humans extend their hips and knees, and plantarflex their ankles to a greater degree than chimpanzees during the second double-support phase of stance^38^,^39^, resulting in feet that are positioned at a greater angle to the ground during push off. This drives the human MTPJs into higher passive dorsiflexion angles, which tightens the plantar aponeurosis and helps to convert ankle power into propulsive force^22^. Chimpanzees possess a far less developed plantar aponeurosis than humans^1^, so greater dorsiflexion of the MTPJs will not stabilize the midfoot and facilitate propulsion as in humans.

The interspecies differences in MTPJ kinematics revealed in this study can be related back to joint morphology: Compared to chimpanzees, humans possess metatarsal heads that are more dorsally oriented, allowing them to push off with more dorsiflexed MTPJs. The results of this study indicate that this feature is an especially good predictor of hominoid MTPJ function during push off in terrestrial locomotion. The similarity in chimpanzee MTPJ kinematics during bipedal and quadrupedal locomotion bolsters this notion. Chimpanzees used slightly greater dorsiflexion angles at MTPJs 2–5 during quadrupedal locomotion, possibly as a result of more retracted hind limb postures that would increase the angle of the foot with the ground at push off^39^. However, the magnitude of this difference between locomotor modes is small (2–5° on average) and only reaches significance for MTPJ 3. Thus, MT head morphology appears to be a good predictor of chimpanzee MTPJ kinematics across terrestrial locomotor modes.

Our results also strongly support our second prediction, that within species inter-ray differences in MTPJ dorsiflexion angles reflect differences in MT head morphology. Humans exhibit diminishing peak dorsiflexion angles from medial to lateral, which is consistent with the findings of previous studies^3^,^21^. Chimpanzees, on the other hand, exhibit significantly lower peak dorsiflexion angles at MTPJ 1 than at MTPJ 2, but like humans exhibit diminishing peak dorsiflexion angles from MTPJ 2 to 5. The drop-off in dorsiflexion angle magnitude is steeper in chimpanzees, perhaps due to the oblique orientation of their feet relative to the direction of travel during terrestrial locomotion. In this position the hallux is directed anteromedially, and the lateral digits are directed anterolaterally. Push off occurs at the medial forefoot near MT heads 2 and 3^40^,^41^, so the MTPJs further from this location exhibit progressively lower peak dorsiflexion angles and contact pressures. As a result, the lateral MTPJs of chimpanzees require less structural reinforcement dorsally during push off.

The kinematic trend in MTPJs 2–5 in humans and chimpanzees is mirrored in the morphometric PC 2 scores, which reflect relative MT head robusticity (mediolateral breadth). The patterns of statistically significant differences between rays in MTPJ kinematics and MT head robusticity are similar, particularly for chimpanzees. Inter-ray differences in chimpanzee MT head robusticity mirror quadrupedal MTPJ kinematics exactly, and closely track differences in bipedal kinematics as well. The decline in MT head robusticity that runs laterally across the forefoot in humans and chimpanzees likely reflects both relative peak MTPJ dorsiflexion angle and load at push off. The magnitude of the decline between MT 2 and MT 3 is steeper for humans than for chimpanzees, likely due to differences in the position of the foot during walking: Humans push off from an axis between MTPJs 1 and 2^11^, whereas chimpanzees push off from an axis between MTPJs 2 and 3^40^,^41^. Humans use the forefoot’s ‘transverse axis’^11^, which maximizes the foot’s leverage during push off. The chimpanzee push-off axis is aligned more closely with the forefoot’s ‘oblique axis’, which was defined by Bojsen-Møller^11^ as passing through MT heads 2–5. However, the actual push-off axis in chimpanzees is likely to differ from this in passing primarily through MT heads 2 and 3, based on the extremely low dorsiflexion angles measured for chimpanzees at MTPJs 4 and 5.

The kinematic results of this study extend those of Griffin *et al*.^3^ for bonobos, but unlike in that study we found no potential for overlap in peak MTPJ dorsiflexion angles between humans and chimpanzees. In particular the chimpanzees in the present study exhibited markedly lower peak dorsiflexion angles for the first MTPJ than those reported by Griffin *et al*. for bonobos. This discrepancy could reflect an actual interspecific difference, but it is more likely to be the product of methodological differences in kinematic data collection between studies. Griffin *et al*. were unable to place markers on their subjects, which can result in errors associated with estimating joint position from static video frames. In the present study, we applied markers to the feet and digits of our subjects to more rigorously quantify MTPJ position.

The morphometric results for A.L. 333-115 reveal a mixed human-chimpanzee morphological pattern for the MT heads of *A. afarensis*. Specifically, MT1 and MT4 are chimpanzee-like on PC1, MT2 and MT5 are human-like, and MT3 falls intermediate between the human and chimpanzee clusters. The morphology pushing some of the A.L. 333 MTs into the chimpanzee distribution is the narrow, chimpanzee-like epicondylar breadth found on some of the MT heads. This morphology is correlated with dorsal MT head gracility and suggests that some australopithecine MTPJs would not have been as stable as in modern humans during moments of high dorsiflexion. The dorsally gracile MT1 of *A. afarensis* further suggests that this species did not push off from the transverse axis between MTPJs 1 and 2 that is characteristic of human walking^11^. However, *A. afarensis* does possess a more dorsally robust MT2 head, and a morphologically intermediate MT3 head, indicating the presence of a push-off axis between MTPJs 2 and 3. The discrepant signals of the MT 4 and 5 heads for *A. afarensis* are more difficult to interpret. However, lower peak dorsiflexion occurs at MTPJs 4 and 5 in both humans and chimpanzees compared to MTPJs 2 and 3 during push off, and thus the morphology at these joints may be of less importance to fossil locomotor behavior reconstructions.

In sum, these results suggest that the forefoot kinematics of *A. afarensis* during push off in bipedal walking were likely to have been unique, and somewhat intermediate between modern humans and chimpanzees. *A. afarensis* probably pushed off from an axis located near the MT2 and 3 heads, and lacked the joint stability to dorsiflex its MTPJ 1 as much as humans at the end of stance. Such a push-off would accord with morphological studies that have suggested a more mobile and/or divergent hallux in *A. afarensis* than in modern humans^4^,^5^,^42^,^43^, although this notion is contested by other studies of the first tarsometatarsal joint in this species^2^,^44^. It is also unclear if *A. afarensis* possessed a plantar aponeurosis similar to that of humans, but if it did it probably could not have utilized it in propulsive force production and midfoot stabilization as effectively as humans due to reduced MTPJ 1 dorsiflexion during push off. These factors may have resulted in a less efficient form of bipedal locomotion in *A. afarensis* as compared to that of modern humans.

Others, however, have interpreted *A. afarensis* MT head morphology as indicating that this species did in fact use human-like push-off kinematics. In particular, Latimer and Lovejoy^19^ and Ward *et al*.^36^ have argued that the MT heads of this species resemble those of humans in being ‘domed’ dorsally, which would facilitate human-like dorsiflexion angles at push off. However, these inferences were based on qualitative descriptions of joint morphology, whereas the present study used quantitative 3D shape analyses to diagnose a more intermediate morphological affinity of the *A. afarensis* MT heads. In support of our results, Duncan *et al*.^14^ also found that the MTPJ morphology of *A. afarensis* fell between that of humans and chimpanzees based on a multivariate discriminant analysis. Thus, qualitative interpretation of MT head morphology may not be as reliable a mode for reconstructing MTPJ function from fossil hominin material.

One caveat that must be acknowledged is the condition of the MT heads in the A.L. 333-115 fossils. These fossils demonstrate some weathering, but most of the important morphological features are well preserved^45^. Damage to the metatarsal heads is reported in the original descriptions for the plantar aspects of the MT heads, where cancellous bone has been exposed in the plantar condyles of A.L. 333-115B, D and E. Because shape changes associated with plantar condylar morphology explained a comparatively small amount of shape variance (<5%) by PCs not presented here, we believe that the damage to most of these fossils does not significantly impact our results. A.L. 333-115C (the MT 3) is one major exception because a small part of the dorsal articular surface of the MT head has broken off such that the articular surface does not project above the dorsum of the diaphysis as it does in A.L. 333-115B, the MT 2 (Fig. 1). This damage has a probable influence on the PC1 score that may shift the fossil towards the chimpanzee PC1 distribution. If complete, this fossil might actually fall within the human distribution, making it more like the A.L. 333-115B MT 2 specimen. In this case the overall morphology of the *A. afarensis* foot would still be mosaic in the pre- and postaxial pedal rays, which would not diminish our suggestion that *A. afarensis* pushed off from a forefoot axis situated between MT heads 2 and 3.

With regard to the broader significance of this study, no previous investigation of hominoid functional morphology has provided both detailed 3D morphometric and kinematic data to rigorously test postcranial joint form-function relationships. In this study we present marker-based 3D kinematics collected from human and chimpanzee subjects *in vivo* along with modern shape analysis techniques to illustrate the strong relationship between MTPJ articular surface morphology and joint function during terrestrial locomotion. Our results provide a case in which 3DGM shape variables can be used to discern functional aspects of postcranial anatomy, and therefore may be useful in reconstructing gait biomechanics from fossil material. Some have recommended caution when using 3DGM to assess form-function relationships due to the many factors that influence bone shape in addition to mechanical function (e.g., phylogeny, pathology, and ontogeny)^46^. However, this study provides an example of how clearly defined hypotheses and careful experimental design can be implemented to verify functional signals in 3DGM analyses. Previous work has shown that phylogenetic signal was not a significant determinant of MT head shape, at least in the hallux^8^, which provides further support to the notion that the MTPJ morphological signals quantified in this study are related to biomechanical function. Studies that integrate experimental approaches and morphological analysis are rare, yet they are critical to establishing true form-function relationships that can be used in the interpretation of skeletal material, and will ultimately provide researchers with the best tools for understanding the evolution of bipedalism.

## Materials and Methods

### 3D Kinematics

All experimental procedures involving human subjects were approved by Stony Brook University’s Institutional Review Board, and all those involving chimpanzee subjects were approved by Stony Brook University’s Institutional Animal Care and Use Committee. Experiments were carried out in accordance with the approved guidelines, and each human subject provided written and informed consent before participating in an experiment. We collected 3D kinematic data from five male humans (28 ± 1 yrs, 68 ± 6 kg) and two male chimpanzees (both 7–8 yrs, 35–50 kg over the period of data collection) during locomotion on a flat 11 m long runway using a four camera high-speed (150 Hz) motion capture system (Xcitex Inc., Woburn, MA, USA). Prior to data collection we applied 1 cm diameter markers to the legs and feet of our subjects using non-toxic paint. To capture MTPJ motion we applied markers to the skin over the head and base of each metatarsal, and to the skin over the proximal and distal ends of each proximal phalanx (Supplementary Fig. 1). During locomotion experiments, all subjects were recorded while walking at self-selected speeds. Chimpanzee subjects were encouraged to walk bipedally and quadrupedally over the runway by an animal trainer who used a fruit or juice reward.

3D kinematic data were extracted from video recordings using ProAnalyst software (Xcitex, Inc.) and imported into MATLAB software (The Mathworks Inc., Natick, MA, USA) for further analysis. We analyzed 10 bipedal and five quadrupedal steps per chimpanzee subject, and five steps per human subject. The lower sample size for chimpanzee quadrupedal steps is due to our difficulty obtaining videos of steps where the view of the foot was not obstructed by the ipsilateral forelimb. Accurate marker tracking requires the use of zoomed-in camera views, which precluded the measurement of velocity of locomotion. Therefore, we measured velocity in separate trials using zoomed-out camera views, and calculated least-squares linear regression equations for the relationship between stance phase duration and dimensionless walking velocity^38^ for each subject (Supplementary Table 1). We then used these equations to estimate dimensionless velocity for each step selected for analysis. Average estimated dimensionless velocities for all steps were similar between species (0.46 ± 0.05 bipedal chimpanzees; 0.42 ± 0.04 quadrupedal chimpanzees; 0.45 ± 0.02 humans; Supplementary Table 1). Raw coordinate data of kinematic markers were filtered using a fourth order, low pass Butterworth filter with a 5–7 Hz cutoff frequency as determined appropriate by residual analysis^47^. MTPJ dorsiflexion was calculated by using the filtered coordinates for each marker to calculate the position of the metatarsal and proximal phalanx of each ray as segment vectors. Joint angles were calculated for each frame of stance phase using vector algebra.

**Figure 2:**
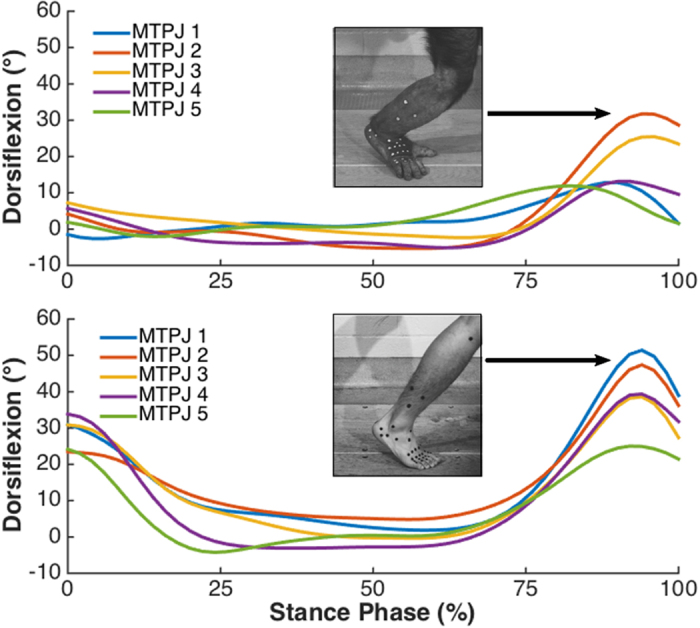
Examples of metatarsophalangeal joint motion during stance phase of bipedal steps from a chimpanzee (top) and a human (bottom). The peak dorsiflexion angles at the end of stance were used in statistical analyses.

**Figure 3:**
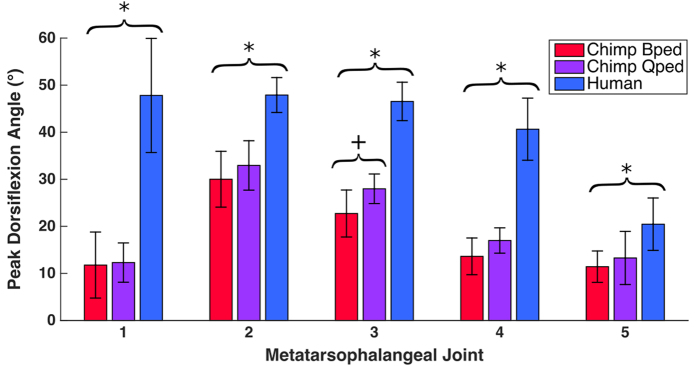
Average peak metatarsophalangeal joint dorsiflexion angles during push off in humans and chimpanzees. Plus/minus one standard deviation is indicated by error bars. *Human angles are significantly different from both bipedal and quadrupedal chimpanzee angles (Mann-Whitney U test, *P* < 0.001). ^+^Chimpanzee bipedal and quadrupedal angles are significantly different from one another (Mann-Whitney U test, *P* = 0.001).

**Figure 4:**
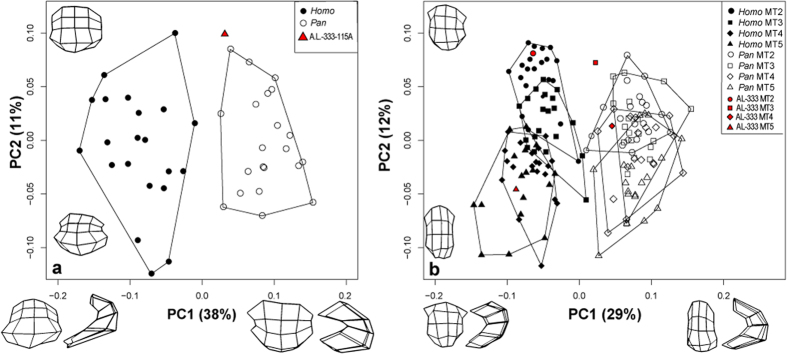
PCA scatterplot of PC1 vs. PC2 for MT1 (left) and MT2–MT5 (right). PC1 tracks dorso-plantar orientation of the MT head whereas PC2 tracks mediolateral MT head breadth. High negative PC scores indicate dorsally oriented MT heads, whereas high positive PC scores indicate more plantarly oriented MT heads. PC2 captures overall MT head robusticity, with considerable overlap between species. Wireframes represent articular shape differences of observed extremes for MT1 (PC1: −0.19–0.16; PC2: −0.10–0.12) and MT2–5 (PC1:−0.12–0.15; PC2: −0.08–0.12).

We assumed that motion at the MTPJs occurs primarily in vertical planes aligned with and perpendicular to the long axes of the digits, and thus the angles that we measured in this manner represent plantarflexion-dorsiflexion. From these angles, we calculated the maximum MTPJ dorsiflexion angle that occurred in the last 15% of stance prior to liftoff (Fig. 2). We used non-parametric Mann-Whitney U tests^48^ to test for differences in MTPJ angles between groups in the following comparisons: human steps *vs.* chimpanzee bipedal steps, human steps *vs*. chimpanzee quadrupedal steps, and chimpanzee bipedal *vs*. quadrupedal steps. We also used non-parametric Kruskal-Wallis tests^48^ to test for differences in MTPJ angles between rays within species. In cases of significant difference, we conducted *post hoc* tests to look for differences between ray pairs. For all Mann-Whitney U and *post-hoc* tests, a sequential Bonferroni correction was applied to the alpha value^49^. All statistical tests involving kinematic data were carried out using MATLAB software.

### 3D Morphometrics

Shape differences in MT1–MT5 were quantified within a comparative sample (n = 25) of *Pan troglodytes* and modern, minimally shod *Homo sapiens* from South Asia. Specimens were sampled from the American Museum of Natural History (AMNH), the Smithsonian’s National Museum of Natural History (USNM), the Cleveland Museum of Natural History (CMNH), and collections housed in the Anatomical Sciences Department at Stony Brook University (SBU). A.L. 333-115A–E surface landmark data were collected from high resolution, research quality casts without any reconstruction housed at the Institute of Human Origins (Arizona State University). The scans were provided by Dr. Caley Orr (Dept. of Cell and Developmental Biology, School of Medicine, University of Colorado – Denver) and Dr. William Kimbel (Institute of Human Origins, Arizona State University). Distal metatarsal morphology was explored using 3D digital polygon models reconstructed from medical computed tomography (CT) and 3D laser surface scans. Measurement error due to data acquisition method is known to be insignificant^50^, so all imaging data were pooled. Scanning was conducted using a NextEngine 3D laser scanner (10,000 pts/inch^2^ resolution per scan, 12 scans per bone) or a General Electric Lightspeed VCT Scanner at the Health Sciences Center, Stony Brook University (120 kV, 250 mA, 1 mm slice thickness, 0.187 mm reconstruction increment, bone plus reconstruction). 3D models from laser scans were merged using ScanStudio HD PRO (NextEngine, Inc) and Geomagic Studio 2012 SR1 (3D Systems, Inc) software packages. 3D models from CT scans were constructed from DICOM data using Amira 5.3.3 software (Visage Imaging, Inc.). Additionally, any scanning artifacts or anomalies in polygonal meshes were corrected in Geomagic and MeshLab 1.3.2 (Visual Computing Lab—ISTI—CNR). All data were collected from right metatarsals whenever possible but some left metatarsals were mirrored in Geomagic when right elements were unavailable.

The 3D shapes of all five metatarsal heads were evaluated using a landmark-based approach. A 5 × 5 3D surface patch of nine user-defined landmarks (Supplementary Table 2) and 16 semi-landmarks was applied using *Landmark Editor*^51^ following the methods detailed in Fernández *et al*.^8^. This landmark set has been shown to be repeatable while also capturing the full articular surface morphology of the metatarsal head. The surface patch was bounded mediolaterally by the proximal termination of the distal epicondyles, dorsally at the proximal termination of the distal articular surface, and plantarly at the proximal termination of the plantar condyles. See previous work for specific anatomical locations of each landmark^8^. Because the hallux differs from the lateral metatarsals in its ontogenetic development and gene expression^37^, it possesses a unique shape that presents possible homology problems with the surface landmarks used in MT2–5. Therefore, MT1 morphometric data were analyzed separately from those of the lesser toes.

Semi-landmarks were slid using the minimized Procrustes distance criterion^52^, but similar results were obtained using minimized bending energy algorithms^53^. The slid coordinates were then subjected to a Generalized Procrustes Analysis (GPA)^54^, and principal component analysis (PCA) was used to summarize and explore the observed variation in metatarsal head shape. Articular surface wireframes and polygonal mesh warps were constructed to visualize shape changes. Significant differences in PC scores between taxonomic groups were analyzed using multivariate analysis of variance (MANOVA); Tukey’s HSD was used for pairwise *post hoc* between-species and between-ray comparisons. All PCs explaining at least 5% of the total shape variance were subjected to these analyses, but because the study sample completely overlapped in all PCs beyond the first two, only the results from PC1 and PC2 are presented. Box-and-whisker plots are shown to visualize the variation within and between species in specific MT head morphologies (Supplementary Fig. 2). Semi-landmark sliding, GPA, PCA, and linear regression analyses were performed in the R package *Geomorph* (ver. 2.17)^55^,^56^. All statistical tests and associated graphics were executed in R 3.2.2 base package (https://www.R-project.org). Wireframe construction and allometric regressions were performed in *MorphoJ* (ver. 1.06b)^57^.

## Additional Information

**How to cite this article**: Fernández, P. J. *et al*. Form and function of the human and chimpanzee forefoot: implications for early hominin bipedalism. *Sci. Rep.* **6**, 30532; doi: 10.1038/srep30532 (2016).

## Supplementary Material

Supplementary Information

## Figures and Tables

**Table 1:** Average ± SD peak metatarsophalangeal joint (MTPJ) dorsiflexion angles during push off in humans and chimpanzees.

MTPJ	Human (N = 25)	Chimp Biped (N = 20)	Chimp Quadruped (N = 10)
1	48 ± 12°*	12 ± 7°	12 ± 4°
2	48 ± 4°*	30 ± 6°	33 ± 5°
3	47 ± 4°*	23 ± 5°§	28 ± 3°
4	41 ± 7°*	14 ± 4°	17 ± 3°
5	21 ± 6°*	11 ± 3°	13 ± 6°

^*^Significant difference between human and chimpanzee (bipedal *and* quadrupedal) angles (*P* < 0.001).

^§^Significant difference between chimpanzee bipedal and chimpanzee quadrupedal angles (*P* < 0.003).

**Table 2:** *Post hoc* pairwise comparisons of peak metatarsophalangeal joint (MTPJ) angles and metatarsal (MT) head PC2 scores between pedal rays in humans and chimpanzees.

Pedal Rays Compared	Human		Chimpanzee Biped		Chimpanzee Quadruped	
	MTPJ Angles*	MT Head PC2§	MTPJ Angles*	MT Head PC2§	MTPJ Angles*	MT Head PC2§
1 vs. 2	n.s.	—	2 > 1 (*P* < 0.001)	—	2 > 1 (*P* < 0.001)	—
1 vs. 3	n.s	—	3 > 1 (*P* < 0.001)	—	3 > 1 (*P* < 0.001)	—
1 vs. 4	n.s.	—	n.s.	—	n.s.	—
1 vs. 5	1 > 5 (*P* < 0.001)	—	n.s.	—	n.s.	—
2 vs. 3	n.s.	2 > 3 (*P* < 0.001)	n.s.	n.s.	n.s.	n.s.
2 vs. 4	n.s.	2 > 4 (*P* < 0.001)	2 > 4 (*P* < 0.001)	2 > 4 (*P* < 0.001)	2 > 4 (*P* = 0.006)	2 > 4 (*P* < 0.001)
2 vs. 5	2 > 5 (*P* < 0.001)	2 > 5 (*P* < 0.001)	2 > 5 (*P* < 0.001)	2 > 5 (*P* < 0.001)	2 > 5 (*P* < 0.001)	2 > 5 (*P* < 0.001)
3 vs. 4	n.s.	3 > 4 (*P* < 0.001)	3 > 4 (*P* = 0.002)	n.s.	n.s.	n.s.
3 vs. 5	3 > 5 (*P* < 0.001)	3 > 5 (*P* < 0.001)	3 > 5 (*P* < 0.001)	3 > 5 (*P* < 0.001)	3 > 5 (*P* < 0.001)	3 > 5 (*P* < 0.001)
4 vs. 5	4 > 5 (*P* < 0.001)	n.s.	n.s.	n.s.	n.s.	n.s.

^*^Significant difference between peak dorsiflexion angles of MTPJs based on Kruskal-Wallis *post hoc* tests with a sequential Bonferroni correction.

^§^Significant difference in MT head PC2 scores based on Tukey’s HSD *post hoc* tests. No comparisons involving the head of MT 1 were performed, because non-homologous landmarks were used to capture the shape of this element that were not used for heads of MTs 2–5.
