# Supplementary material for: Form and function of the human and chimpanzee forefoot: implications for early hominin bipedalism
Source: Sci Rep. 2016 Jul 28;6:30532. doi: 10.1038/srep30532 (PMC4964565; doi:10.1038/srep30532)
Supplement: Supplementary Information [file srep30532-s1.pdf]

Form and function of the human and chimpanzee forefoot: implications for early hominin bipedalism

Peter J. Fernández, Nicholas B. Holowka, Brigitte Demes, William L. Jungers

Additional Methodology

Speed estimation. Due to the density of markers that we used to capture foot kinematics we had to use zoomed-in camera views that precluded the measurement of velocity of progression for complete strides. Therefore, we measured velocity (v_d) in separate trials using zoomed-out camera views as the anteroposterior distance traveled by a marker placed on the pelvis (anterior superior iliac spine or posterior superior iliac spine) over the duration of a stride. We also measured hip height (L) at 50% of stance using a marker placed over the greater trochanter, and used these variables to calculate dimensionless velocity (v) following the equation used by O'Neill et al.[1]:

$$v = v_d/(g * L)^{1/2}$$

where g is the gravitational constant 9.81 ms^{-2}. We measured stance phase duration in each of these trials, and calculated least-squares linear regression equations for the relationship between natural logs of the inverse of stance duration and dimensionless velocity for each subject (Supplementary Table 1). We then used these equations to estimate dimensionless velocity for each step selected for analysis.

1 O'Neill, M. C. *et al.* Three-dimensional kinematics of the pelvis and hind limbs in chimpanzee (*Pan troglodytes*) and human bipedal walking. *J. Hum. Evol.* **86**, 32-42, (2015).

Supplementary Table 1. Least-squares linear regression equations describing the relationship between dimensionless velocity (*v*) and stance phase duration (t_s) for each subject, and average t_s measured and *v* estimated from all steps analyzed for each subject. See preceding text for description of equation. *Standard error of estimate.

Subject	Equation	SEE*	R^2	t_s (s)	Estimated *v*
Chimpanzee Biped					
A	$\ln(v) = 0.76*\ln(t_s^{-1}) - 1.25$	0.034	0.78	0.6 ± 0.06	0.42 ± 0.03
B	$\ln(v) = 0.79*\ln(t_s^{-1}) - 1.17$	0.03	0.72	0.58 ± 0.06	0.48 ± 0.02
Average				*0.58 ± 0.05*	*0.46 ± 0.05*
Chimpanzee Quadruped					
A	$\ln(v) = 1.41*\ln(t_s^{-1}) - 1.39$	0.11	0.73	0.68 ± 0.02	0.43 ± 0.02
B	$\ln(v) = 1.37*\ln(t_s^{-1}) - 1.34$	0.071	0.91	0.73 ± 0.08	0.41 ± 0.05
Average				*0.71 ± 0.06*	*0.42 ± 0.04*
Human					
A	$\ln(v) = 1.54*\ln(t_s^{-1}) - 1.59$	0.033	0.98	0.59 ± 0.01	0.46 ± 0.01
B	$\ln(v) = 2.07*\ln(t_s^{-1}) - 1.73$	0.039	0.95	0.65 ± 0.01	0.43 ± 0.01
C	$\ln(v) = 1.63*\ln(t_s^{-1}) - 1.42$	0.027	0.98	0.69 ± 0.02	0.44 ± 0.02
D	$\ln(v) = 1.47*\ln(t_s^{-1}) - 1.36$	0.046	0.96	0.66 ± 0.01	0.47 ± 0.01
E	$\ln(v) = 1.85*\ln(t_s^{-1}) - 1.47$	0.053	0.96	0.68 ± 0.01	0.47 ± 0.01
Average				*0.66 ± 0.04*	*0.45 ± 0.02*

Landmark Number	Type	Landmark Description
1	Type II	most medially protruding point on medial epicondyle
2	Type III	midpoint between point 1 and 3, medial ridge separating distal articular surface from MT diaphysis
3	Type II	most plantarly projecting point on medial plantar condyle
4	Type II	dorsal most point on dorsal surface of MT articular surface
5	Type II	most distally projecting point on MT head
6	Type III	most plantarly projecting point on intercondylar ridge
7	Type II	most laterally protruding point on lateral epicondyle
8	Type III	midpoint between point 7 and 9, lateral ridge separating distal articular surface from MT diaphysis
9	Type II	most plantarly projecting point on lateral plantar condyle

Supplementary Table 2. List of landmarks on each metatarsal head. Semilandmark locations are calculated using minimized Procrustes distance between reference landmarks. Refer to Figure 2 in Fernández et al.[2] for semilandmark location placement on the distal MT articular surface.

2 Fernández, P. J. *et al.* Functional aspects of metatarsal head shape in humans, apes, and Old World monkeys. *J. Hum. Evol.* **86**, 136-146, (2015).

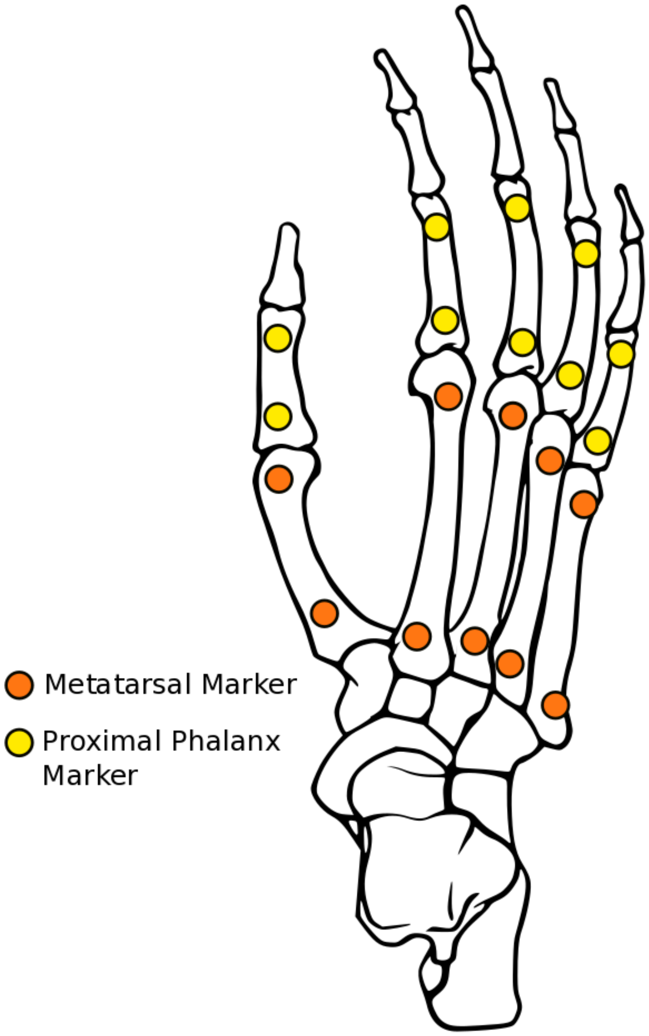

Supplementary Figure 1. Locations for kinematic markers used to measure metatarsophalangeal joint dorsiflexion in all subjects. Chimpanzee foot skeleton redrawn from Morton[3].

3 Morton, D.J. Evolution of the human foot I. *Am. Phys. Anthropol.* **5**, 305-336, (1922).

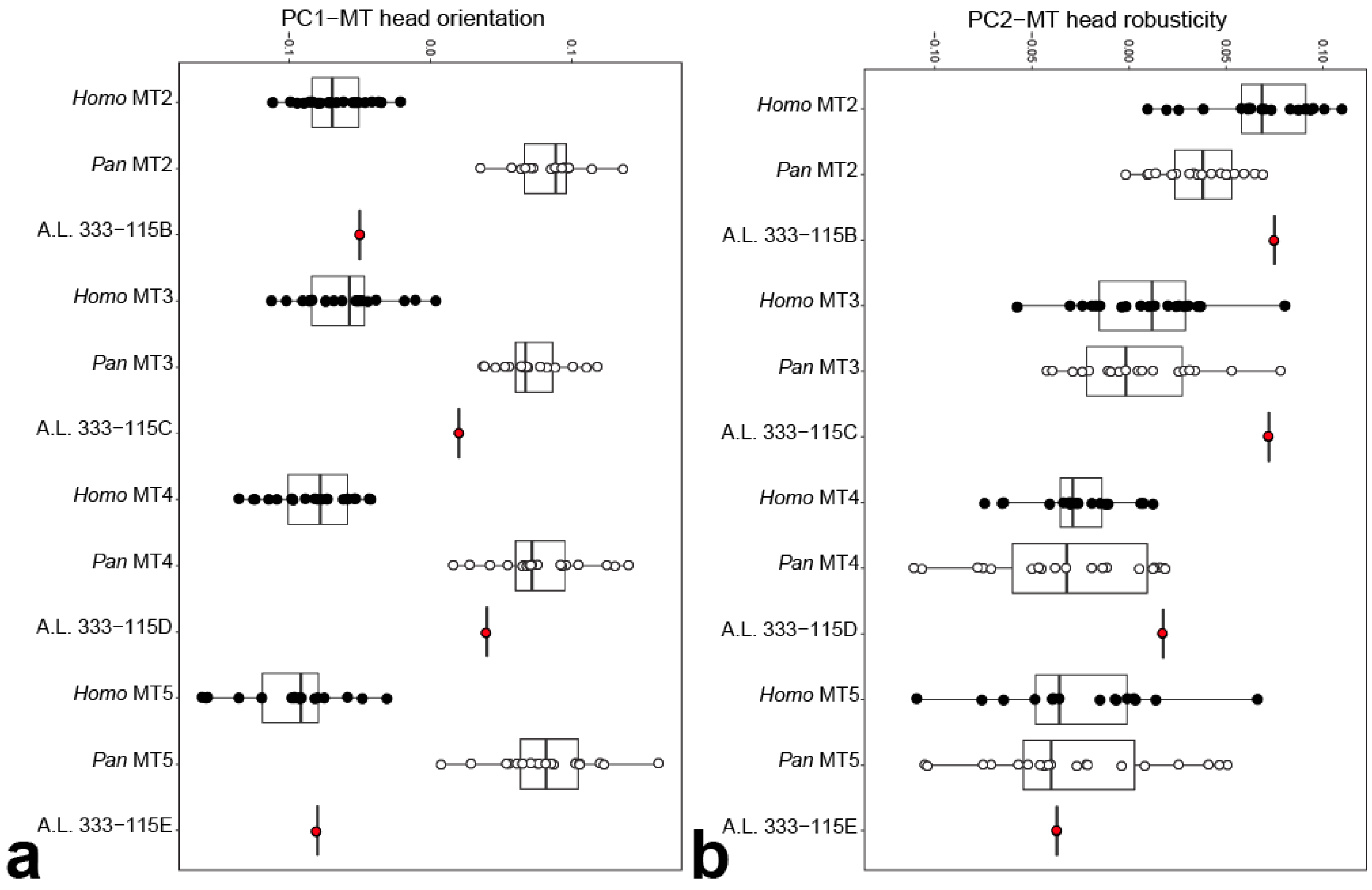

Supplementary Figure 2. Cleveland box-and-whisker plots of PC1 (a) and PC2 (b) scores for MT2-MT5 in humans, chimpanzees, and *Au*, *afarensis*. PC1 tracked overall dorsoplantar MT head orientation and PC2 tracked overall MT head robusticity. For AL-333-115, the MT2 is human-like on PC1 and PC2, the MT3 is intermediate on PC1 and within the chimpanzee and human range on PC2, the MT4 is human-like on PC1 and PC2, and the MT5 is human-like on PC1 while overlapping both species on PC2. Vertical bars: median. Rectangles: interquartile range. Horizontal bars: range.
